# Identification and Characterization of *LARGE EMBRYO*, a New Gene Controlling Embryo Size in Rice (*Oryza sativa* L.)

**DOI:** 10.1186/s12284-019-0277-y

**Published:** 2019-04-11

**Authors:** Gileung Lee, Rihua Piao, Yunjoo Lee, Backki Kim, Jeonghwan Seo, Dongryung Lee, Su Jang, Zhuo Jin, Choonseok Lee, Joong Hyoun Chin, Hee-Jong Koh

**Affiliations:** 1 Department of Plant Science and Research Institute for Agriculture and Life Sciences, and Plant Genomics and Breeding Institute, Seoul National University, Seoul, 08826 South Korea; 2 Rice Research Institute, Jilin Academy of Agricultural Sciences, Gongzhuling, 136100 Jilin China; 3 Graduate School of Integrated Bioindustry, Sejong University, 209, Neungdong-ro, Gwangjin-gu, Seoul 05006 South Korea

**Keywords:** Rice, Large embryo, Giant embryo, C3HC4-type RING finger protein, Nutritional value

## Abstract

**Background:**

Although embryo accounts for only 2–3% of the total weight of a rice grain, it is a good source of various nutrients for human health. Because enlarged embryo size causes increase of the amount of nutrients and bioactive compounds stored within rice grain, giant embryo mutants of rice (*Oryza sativa* L.) are excellent genetic resources for improving the nutritional value of rice grains.

**Results:**

Three giant embryo mutants, including *large embryo* (*le*), *giant embryo* (*ge*) and *super*-*giant embryo* (*ge*^*s*^), with variable embryo size were used in this study. We investigated whether genes controlling embryo size in these mutants (*le*, *ge* and *ge*^*s*^) were allelic to each other. Although *ge* and *ge*^*s*^ was allelic to *GIANT EMBRY* (*GE*), *le* was not allelic to *ge* and *ge*^*s*^ in allelism test. The *GE* gene carried a unique nucleotide substitution in each of the two mutants (*ge* and *ge*^*s*^), resulting in non-synonymous mutations in exon 2 of *GE* in both mutants. However, the *GE* gene of the *le* mutant did not carry any mutation, suggesting that the enlarged embryo phenotype of *le* was governed by another gene. Using map-based cloning, we mapped the *LE* gene to the short arm of chromosome 3. The *le* mutant showed mild enlargement in embryo size, which resulted from an increase in the size of scutellar parenchyma cells. The *LE* encodes a C3HC4-type RING finger protein and was expressed to relatively high levels in seeds at a late developmental stage. Knockdown of *LE* expression using RNA interference increased the embryo size of rice grains, confirming the role of *LE* in determining the embryo size.

**Conclusion:**

Overall, we identified a new gene controlling embryo size in rice. Phenotypic and molecular characterization results suggest that the *le* mutant will serve as a valuable resource for developing new rice cultivars with large embryos and nutrient-dense grains.

**Electronic supplementary material:**

The online version of this article (10.1186/s12284-019-0277-y) contains supplementary material, which is available to authorized users.

## Background

With an ever-increasing demand for high quality rice (*Oryza sativa* L.), several attempts have been made to improve the quality of rice (Fitzgerald et al. 2009). A mature rice embryo typically accounts for only 2–3% of the total kernel weight, but has enriched various nutrients, such as proteins, lipids, vitamins, minerals, and phytochemicals (Gopala Krishna et al. 1984; Tanaka et al. 1977). The high nutritional content of embryo makes it popular to use brown rice, polished embryonic rice, and byproduct (bran) generated after polishing.

Breeding efforts to improve the nutritional quality of rice have focused on the induction and isolation of mutants. Various mutants with distinct embryo and endosperm phenotypes have been reported in rice, including endospermless, embryoless, reduced embryo, and giant embryo mutants (Satoh and Omura 1981; Hong et al. 1995). Among these, the giant embryo mutants have often been used as breeding materials to improve the nutritional value of rice. To date, several giant embryo rice cultivars have been reported (Maeda et al. 2001; Takahashi et al. 2009; Hong et al. 2012; Han et al. 2012). Brown rice of these rice mutants contain higher amounts of proteins, lipids, essential amino acids, vitamins (B_1_, B_2_, and E), minerals (Ca, Fe, Mg, K, and P), and bioactive compounds [γ-aminobutyric acid (GABA) and γ-oryzanol] than those of wild-type rice cultivars with normal embryo size (Koh et al. 1993; Zhang et al. 2005; Jeng et al. 2012; Kim et al. 2013).

Until now, only two genes related to the enlargement of embryo size have been identified in rice. *GIANT EMBRYO* (*GE*) was mapped on chromosome 7 (Satoh and Iwata 1990; Koh et al. 1996), and Nagasawa et al. (2013) revealed that *GE* gene encodes CYP78A13, a cytochrome P450 protein, and functions in determining embryo/endosperm size. The loss-of-function *GE* exhibits an enlargement of embryo and shrinkage of endosperm. Additionally, *GE* plays critical role in shoot apical meristem (SAM) maintenance, and *GE* overexpression promotes cell proliferation, plant growth and grain yield (Yang et al. 2013). In maize, the insertion of a transposable element in *ZmGE2*, a homolog of *OsGE*, has resulted in a reduction in its expression and an increase in the embryo to endosperm ratio (EER) (Zhang et al. 2012). *ZmGE2* encodes a cytochrome P450 protein of the CYP78A family, and overexpression of *OsGE* in maize results in a reduction in EER. These results suggest that cytochrome P450 proteins play an important role in regulating embryo size, and the mechanism of EER determination is evolutionarily conserved in rice and maize (Zhang et al. 2012). *GOLIATH* (*GO*), another gene related to embryo size, has been mapped to the long arm of chromosome 3 (Taramino et al. 2003). Independently identified mutant, *plastochron3* (*PLA3*) is allelic to *go* mutant. The loss of function *PLA3*, which encodes glutamate carboxypeptidase, exhibits pleiotropic phenotypes including enlarged embryo (caused by an increase in the number of cells), seed vivipary, defects in SAM maintenance, aberrant leaf morphology (Kawakatsu et al. 2009). Recently, new giant embryo breeding materials have been reported; these display an enlarged embryo and higher content of triacylglycerol compared with the wild type. The gene responsible for these phenotypes is not allelic to *GE* and maps to the short arm of chromosome 3 (Sakata et al. 2016).

In this study, we performed allelism tests to determine if genes responsible for the enlarged embryo phenotypes of the embryo mutants, *ge*, *ge*^*s*^, and *le*, are allelic to each other and identified a new gene controlling embryo size. Our results will contribute to understanding genetic regulation of embryo size. Furthermore, the series of mutants may facilitate the breeding of embryonic cultivar with high nutritional value.

## Results

### Characterization of Three Giant Embryo Rice Mutants

**Fig. 1:**
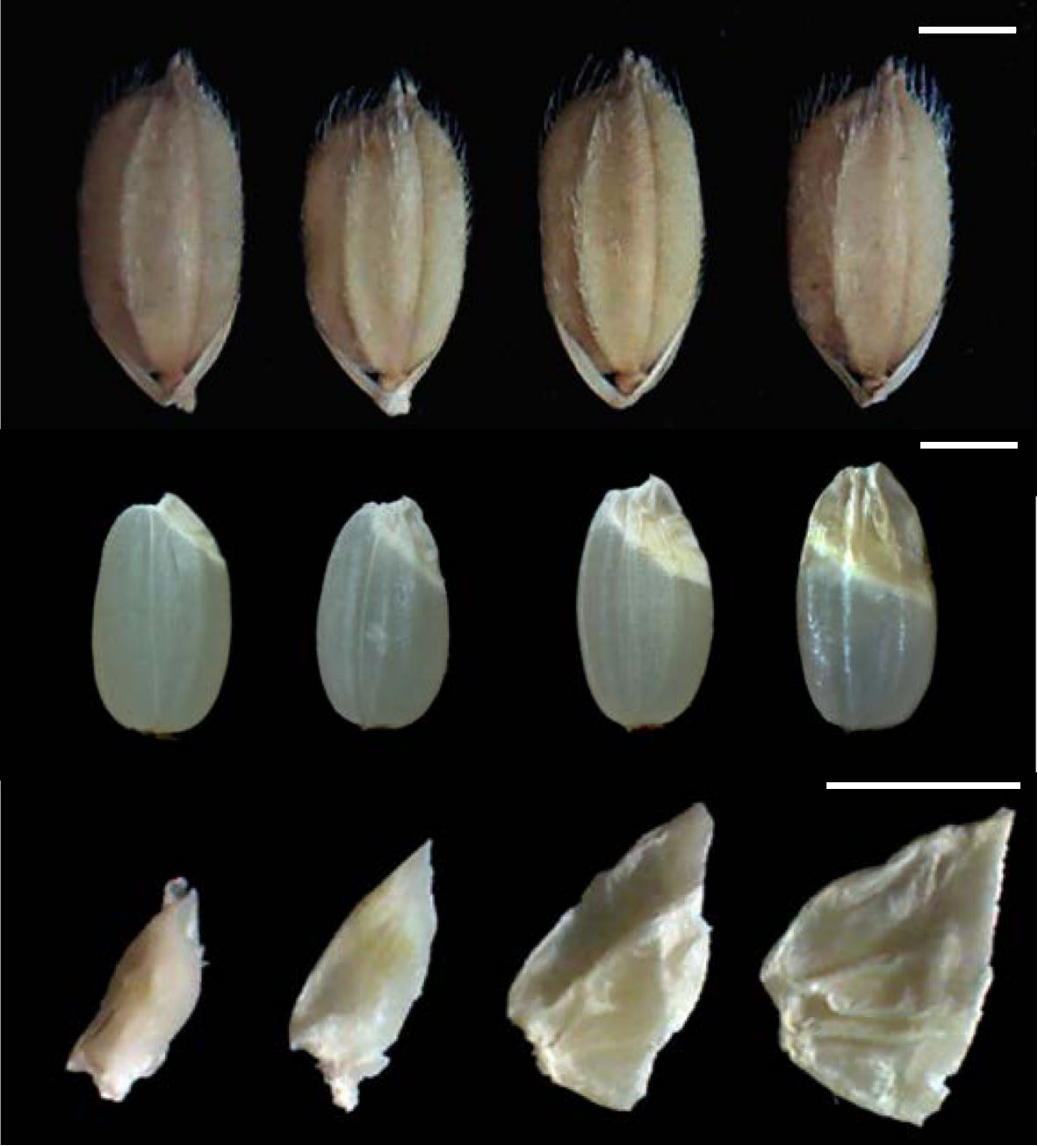
Grain and embryo phenotypes of HC and giant embryo mutants. Images from left to right are of HC, *le*, *ge*, and *ge*^*s*^ in this order. Scale bars = 2 mm

**Fig. 2:**
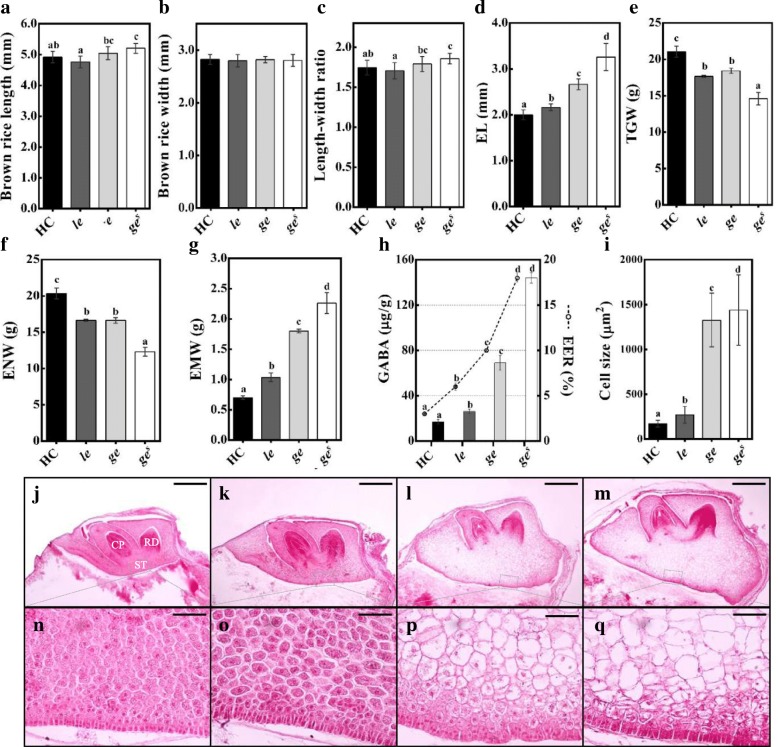
Characterization of phenotypic traits of rice grains and embryos of HC and giant embryo mutants. **a-i** Evalucation of various seed characters. Brown rice length (**a**), width (**b**), and LWR (**c**); Embryo length (EL) (**d**); Thousand-grain weight (TGW) (**e**); Endosperm weight (ENW, 1000 seeds) (**f**); Embryo weight (EMW, 1000 seeds) (**g**); GABA content of EER (**h**); Size of scutellar parenchyma cells (**i**). **j-q** Longitudinal sections of embryo at 20 days after pollination (DAP): wild type (**j** and **n**), *le* (**k** and **o**), *ge* (**l** and **p**), and *ge*^*s*^ (**m** and **q**). CP, coleoptiles; RD, radicle; ST, scutellum (**n**-**q**). The means followed by lowercase letters indicate statistically significant difference (*p* < 0.05, ANOVA) according to Duncan test. Error bars indicate ± s.d. of replicates. Scale bars = 0.5 mm (**j**-**m**), Scale bar = 50 μm (**n**-**q**)

All three mutants exhibited enlarged embryo phenotype compared with Hwacheong (HC). Depending on the embryo size, the three giant embryo mutants were designated as *le*, *ge*, and *ge*^*s*^; the embryo size was relatively smaller in *le*, larger in *ge*^*s*^, and intermediate in *ge* mutant (Figs. 1 and 2d). We evaluated the relative embryo size of these mutants using the EER, which is defined as the weight of the embryo relative to that of the endosperm. The EER gradually increased in the order of *le*, *ge*, and *ge*^*s*^ mutants, and the GABA content of brown rice followed the same trend (Fig. 2h). The enlarged embryo phenotype of all mutants resulted from a conspicuous enlargement of scutellar parenchyma cells (Fig. 2i and n-q). In addition to the embryo size, other phenotypes were also investigated. Although none of the giant embryo mutants showed any major defects in shoot and radicle differentiation, the shoot size of *ge* and *ge*^*s*^ mutants was slightly smaller than that of *le* mutant and wild-type HC (Fig. 2j-m). Among grain characteristics, the width of mutant rice grains was comparable to that of HC; however, the length and length to width ratio (LWR) of the *ge*^*s*^ grains were significantly higher than those of HC (Fig. 2a-c). The giant embryo mutants showed significantly lower thousand-grain weight (TGW) and endosperm weight (ENW) but higher embryo weight (EMW) than HC (Fig. 2e-g). Compared with HC, the *ge* and *ge*^*s*^ mutants showed a dramatic increase in EMW of 157% and 223%, respectively, whereas the *le* mutant exhibited only a 48% increase in EMW (Fig. 2g). Among other agronomic traits, culm length (CL), grain width (GW), grain length (GL), and LWR of the *le* mutant were significantly different from those of HC (Additional file 3: Figure S2).

### Genetic Analysis of Giant Embryo Mutants

In order to confirm allelism and heredity among genes responsible for enlarged embryo phenotype in three mutants, we investigated the segregation ratio of F_2_ seeds derived from reciprocal crosses among HC, *le*, *ge*, and *ge*^*s*^ (Table 1). Data showed that the embryo phenotype of F_2_ seeds derived from reciprocal crosses between each mutant and HC segregated in a 3: 1 ratio (normal: enlarged), indicating that the enlarged embryo phenotype of each mutant was controlled by a single recessive gene. Because we previously showed that *ge*^*s*^ is allelic to *ge* (Kim et al. 1992), we inferred that the enlarged embryo phenotype of *ge*^*s*^ mutant was induced by a mutation in *GIANT EMBRYO* (*GE*) gene. In reciprocal crosses between *ge* and *ge*^*s*^, the EL of F_1_ seeds was increased, and embryos of all F_2_ seeds showed the enlarged phenotype, suggesting that the *ge* locus is identical to that of *ge*^*s*^ (Table 1). On the other hand, F_2_ seeds derived from reciprocal crosses between *le* and each of the other two giant embryo mutants, *ge* and *ge*^*s*^, showed three different embryo phenotypes with 9:3:4 segregation ratio (normal type: *le* type: *ge*/*ge*^*s*^ type) (Additional file 4: Figure S3), suggesting that *LE* is not allelic to *GE*.

**Table 1:** Segregation of F_2_ seeds derived from the reciprocal crosses among HC and three giant embryo mutants

Cross combination	EL of F_1_ seed (mm)	Number of different embryo type				Expected ratio	*x* ^*2*^	*p*-value
		nor	*le*	*ge*	*ge* ^*s*^			
HC/*le*	2.10^a^	299	99	–	–	3:1	0	1
*le*/HC		260	100	–	–	3:1	1.34	0.25
HC/*ge*	2.16^a^	348	–	134	–	3:1	1.87	0.17
*ge*/HC		269	–	83	–	3:1	0.31	0.58
HC/*ge*^*s*^	2.12^a^	246	–	–	76	3:1	0.27	0.61
*ge*^*s*^/HC		265	–	–	84	3:1	0.12	0.73
*le*/*ge*	2.15^a^	177	64	71	–	9:3:4	1.16	0.56
*ge*/*le*		145	48	63	–	9:3:4	0.02	0.99
*le*/*ge*^*s*^	2.21^a^	178	60	–	78	9:3:4	0.02	0.99
*ge*^*s*^/*le*		160	51	–	63	9:3:4	0.67	0.72
*ge*^*s*^/*ge*	3.12^b^	–	–	296	117	3:1	2.27	0.13
*ge*/*ge*^*s*^		–	–	106	45	3:1	1.61	0.2

The means followed by lowercase letters are significantly different (*p* < 0.05, ANOVA) according to Duncan test. *EL* embryo length, *nor* normal type

### Sequencing Analysis of *GE* Locus in the Three Giant Embryo Mutants

**Fig. 3:**
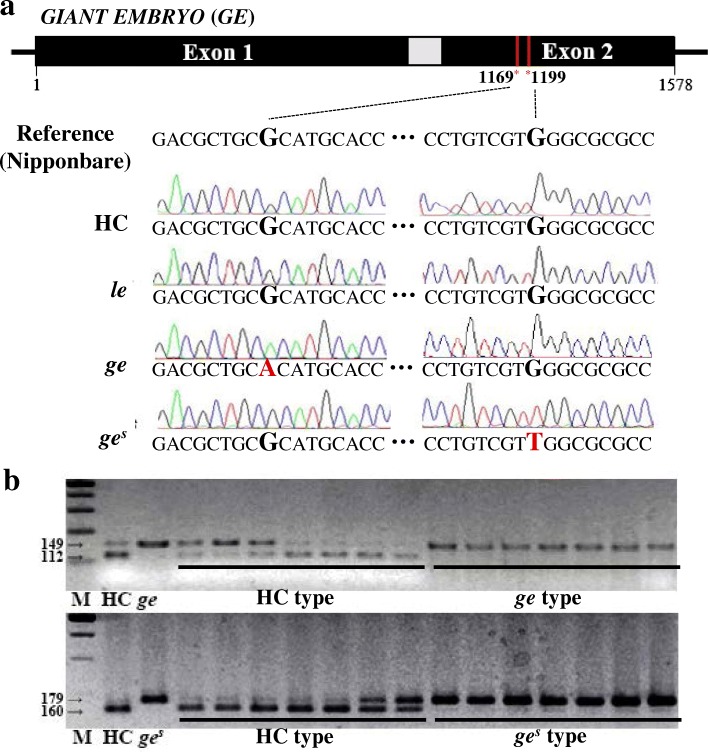
Sequence analysis of the *GE* locus. **a** Identification of mutations in *GE* locus. **b** Co-segregation test of mutation point and enlarged embryo phenotype in F_2_ seeds derived from HC/*ge* and HC/*ge*^*s*^ crosses

We sequenced the *GE* locus (*Os07g0603700*) of all three giant embryo mutants. Data revealed G to A single base change at nucleotide position 1169 in the *GE* locus of *ge* mutant, resulting in arginine (Arg) to histidine (His) amino acid substitution at position 390, and G to T single base change at nucleotide position 1199 in the *GE* locus of *ge*^*s*^ mutant, resulting in tryptophan (Trp) to leucine (Leu) amino acid substitution at position 400 (Fig. 3a). These mutations co-segregated with the enlarged embryo phenotype in F_2_ seeds derived from crosses between HC and each of these two mutants (*ge* and *ge*^*s*^) (Fig. 3b). However, no nucleotide substitutions were detected in the *GE* locus of *le* mutant. Additionally, no genetic polymorphism was present in the *PLA3/GO* locus of *le* mutant (data not shown). These data suggest that, while mutations in *GE* were responsible for the enlarged embryo phenotypes of *ge* and *ge*^*s*^ mutants, another gene was responsible for the enlarged embryo phenotype of *le* mutant. These results were consistent with those of our allelism tests described above.

### Map-Based Cloning of *LE* Gene

**Fig. 4:**
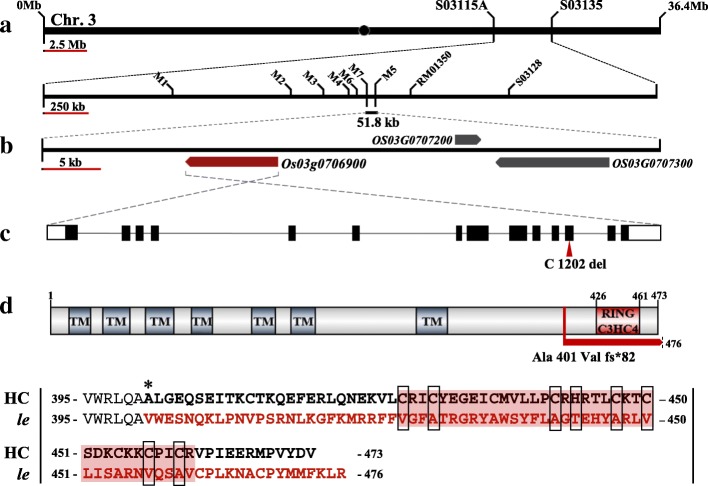
Map based cloning of the *LE* gene. **a** Schematic diagram of mapping of *LE* locus. **b** Candidate ORFs in delimited candidate region. **c** *LE* gene structure and splicing pattern. Black lines, white solid boxes, and black solid boxes indicate intron, UTRs, and exons, respectively. The mutation position is marked with red arrow head. **d** LE protein structure and comparison of amino acid sequence of the frameshift region between the HC and *le* mutant. Asterisk represents the location of amino acid substitution caused by one base deletion in *le* mutant. Amino acids in the region of frameshift (fs) are indicated in red. Red background and black open boxes indicate amino acid sequences of RING C3HC4 domain and conserved residues in the domain

To isolate gene responsible for the enlarged embryo phenotype of *le* mutant, we performed map-based cloning using F_2_ and F_3_ populations derived from a cross between *le* mutant and rice cv. Hangangchal1 (*Tongil*-type). Bulked segregant analysis (BSA) showed that the *LE* gene was linked with the InDel marker S03128 on the short arm of chromosome 3. To determine the exact position of *LE* locus, we developed a new set of InDel markers on the basis of genetic polymorphisms between sequences of *indica* and *japonica* rice (http://www.ncbi.nlm.nih.gov/). Using these markers, *LE* was mapped to an approximately 52-kb region between M7 and M5 markers (Fig. 4a). Three predicted open reading frames (ORFs) were located within this candidate region (Fig. 4b), all of which were sequenced in the *le* mutant and HC. Sequence analysis revealed the deletion of a single nucleotide ‘C’ at position 1202 in exon 12 of the locus *Os03g0706900* in *le* mutant (Fig. 4c). This locus encodes a C3HC4-type RING finger protein. The deletion of ‘C’ at position 1202 was predicted to cause frameshift mutation and termination of the ORF after 82 aberrant amino acids (Fig. 4d and Additional file 5).

To perform co-segregation analysis, we developed a derived cleaved amplified polymorphic sequences (dCAPS) marker. The dCAPS genotype of F_2_ individuals co-segregated with the enlarged embryo phenotype of the F_3_ seeds type. These data showed that the deletion of C base at nucleotide position 1202 of the locus *Os03g0706900* was responsible for the enlarged embryo phenotype of *le* mutant (Additional file 6). To determine whether this mutant variation is present as a natural variation in the rice germplasm, we genotyped the *Os03g0706900* locus in 12 *O. sativa* cultivars (six *japonica*, two *tongil-type*, and four *indica*) and one *O. nivara* accession using the dCAPS marker. However, none of these rice genotypes harbored the mutation present in the *Os03g0706900* locus of *le* mutant (Additional file 6). Therefore, we focused on *Os03g0706900* as a candidate for *LE*.

### Effect of *LE* Knockdown on Embryo Size

**Fig. 5:**
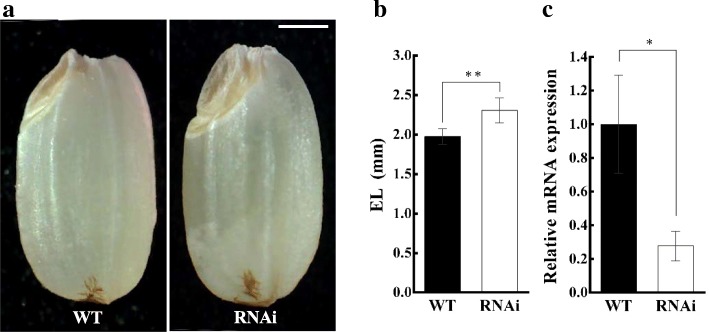
Comparison of embryo phenotype and *LE* expression between RNAi transgenic plant and wild type (Dongjin). **a** Seed phenotype of WT and RNAi plants. **b** Comparison of embryo length between WT and RNAi plants. **c** Relative expression of *LE* (normalized to *UBIQUITIN*) in WT and RNAi plants. Error bars indicate ± s.d. of three replicates. Asterisks represent significant difference, as determined by a two-tailed Student’s *t*-test (*, *p* < 0.05; **, *p* < 0.01). Scale bar = 1 mm

To confirm whether the *LE* gene regulates embryo size in rice, we used dsRNA-mediated interference (RNAi) to knockdown the expression of *LE* in the *japonica* cv. Dongjin. The embryo size of RNAi T_1_-plant grains was significantly bigger than that of wild-type grains (Fig. 5a and c). Quantitative real time PCR (qRT PCR) analysis using leaf samples revealed that the expression of *LE* gene in RNAi T_1_ plants was significantly lower than that in wild-type (Fig. 5c), indicating that *LE* controls embryo size in rice.

### Expression Pattern of *LE*

**Fig. 6:**
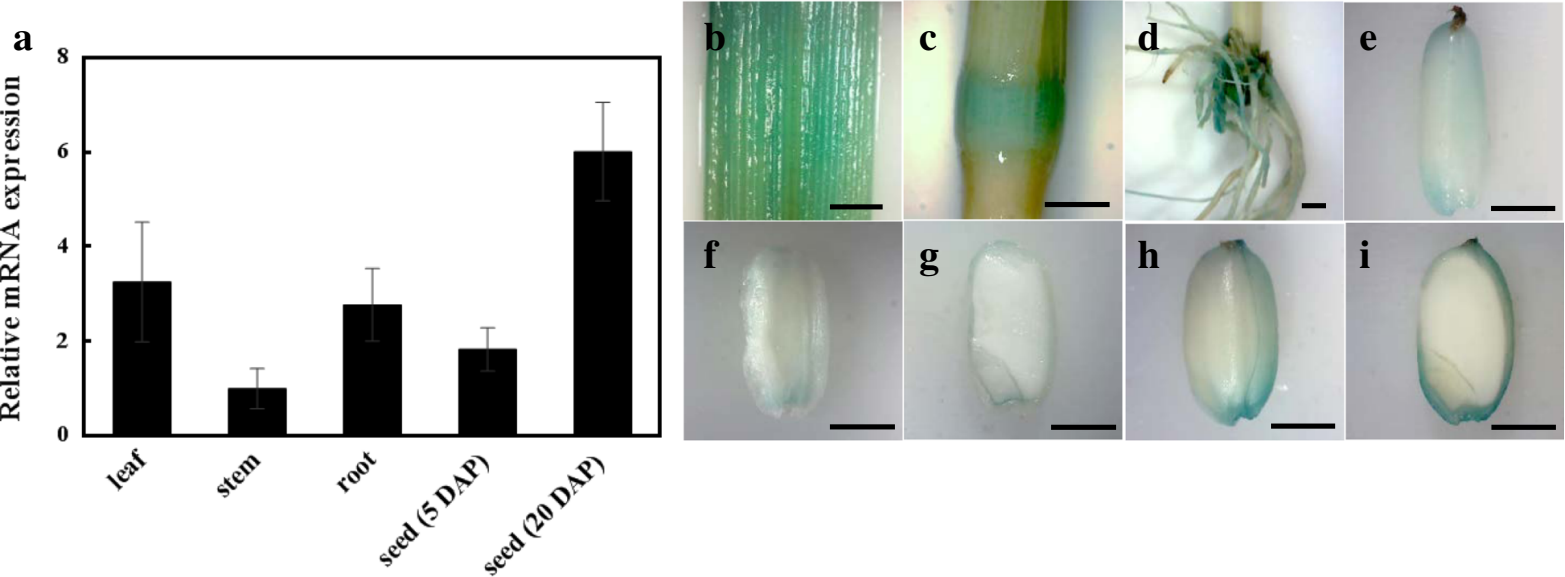
Tissue-specific RNA expression pattern of *LE*. **a** qRT-PCR analysis of *LE* gene in leaf, stem, root and seed (5 and 20 DAP). **b-i*** GUS* expression pattern of various tissues in Pro*LE*:*GUS* transgenic line [leaf (**b**); stem (**c**); root (**d**); seed at 5 DAP (**e**); seed at 10 DAP (**f** and **g**); seed at 20 DAP (**h** and **i**)]. Error bars indicate ± s.d. of replicates. Scale bars = 2 mm

We conducted qRT PCR analysis to monitor the expression of *LE* in various organs, including leaves, stems, roots, and seeds (Fig. 6). *LE* was expressed in all organs in HC (Fig. 6a). The expression of *LE* in stems was lower than that in leaves and roots. In seeds, the expression level of *LE* was much lower at 5 DAP than at 20 DAP, indicating that the expression of *LE* increases as seeds mature (Fig. 6a). We also performed β-glucuronidase (GUS) staining experiments using transgenic rice plants expressing GUS reporter under the control of *LE* gene promoter (*proLE::GUS*). GUS staining data were consistent with the results of qRT PCR analysis (Fig. 6b-i). *GUS* expression was observed in all organs, including leaves, stems, roots, and seeds (Fig. 6b-i), and the expression of *GUS* in seeds increased over time (Fig. 6f-i). Interestingly, GUS staining data showed that *LE* is expressed in the outer part of rice seed (Fig. 6g and i).

## Discussion

In this study, we performed map-based cloning to identify the *LE* gene responsible for the enlarged embryo phenotype of the rice *le* mutant. We showed that *LE* is not allelic to *GE.* Although *LE* was mapped to the same chromosome with *GO* (Kawakatsu et al. 2009) and new *giant embryo* locus (Sakata et al. 2016), the candidate region of *LE* locus was far from the position of *GO* and the other locus. RNAi transgenic approach confirmed that *LE, Os03g0706900,* is the gene responsible for the large embryo in the *le* mutant.

The LE protein consists of 473 amino acid residues with a C3HC4-type RING finger domain at the C-terminus and seven transmembrane motifs at the N-terminus predicted using TMHMM server v. 2.0 (http://www.cbs.dtu.dk/services/TMHMM/) (Additional file 5). Multiple sequence alignment of LE orthologs revealed that the amino acid sequence of the predicted transmembrane motifs and the consensus sequence of RING finger domain (Cys-× _2_-Cys-X_11_-Cys-X-His-X_3_-Cys-× _2_-Cys-X_6_-Cys-X_2_-Cys) were highly conserved across monocots and dicots. However, the consensus sequence of LE in *le* mutant was highly dissimilar because of the frameshift generated by the single nucleotide deletion. (Fig. 4d and Additional file 5). These results indicate that the functional role of LE orthologs is conserved in plants and is dependent on the C3HC4-type RING finger domain. The sequence of *LE* is identical to that of *OsRHC1* cloned and characterized previously (Cheung et al. 2007). *OsRHC1* is differentially expressed in bacterial blight resistant near-isogenic lines (NILs) containing *Xa14* or *Xa23* as well as in NILs susceptible to bacterial blight (Cheung et al. 2007). Additionally, transgenic Arabidopsis plants overexpressing *OsRHC1* are more resistant to bacterial pathogens than wild-type Arabidopsis (Ma et al. 2009). In rice, the function of C3HC4-type RING finger proteins is not well understood. To date, only a few C3HC4-type RING finger proteins have been reported in rice, including *Os*COP1, *Os*COIN1, and *Os*XB3; these are involved in photomorphogenesis, abiotic stress tolerance, and disease resistance, respectively (Tsuge et al. 2001; Liu et al. 2007; Wang et al. 2006). However, there is no report that C3HC4-type RING finger protein is involved in embryo morphology and development in rice. By contrast, the function of C3HC4-type RING finger proteins in Arabidopsis is well characterized, and several genes encoding C3HC4-type RING finger proteins have been identified, including *At*COP1 and *At*CIP8 (photomorphogenesis) (von Arnim and Deng 1993; Hardtke et al. 2002), *At*TED3 (light signaling) (Pepper and Chory 1997), *At*PEX10 *and At*PEX12 (peroxisome biogenesis) (Schumann et al. 2003; Fan et al. 2005), *At*XBAT32 (root development), and *At*SDIR1 (stress tolerance) (Zhang et al. 2007). Among these, *At*PEX10 and *At*PEX12 contain two transmembrane helices and a C3HC4-type RING finger domain, which are similar in sequence to those of *LE*. Both *At*PEX10 and *At*PEX12 are required for peroxisome biogenesis, and their dysfunction leads to lethality at the heart stage of embryogenesis.

Both transmembrane motifs at the N-terminus and a C3HC4-type RING domain at the C-terminus predicted were considered essential for the function and localization of LE protein. Amino acid sequences of *LE* orthologs were highly conserved among monocots and dicots. *ZmZF13*, a maize ortholog of *OsLE*, has been previously identified from a cDNA library obtained from developing maize seeds (Wang et al. 2010). There is, however, no published information on the functions of these *LE* orthologs, except for pathogen defense of *OsRHC1*. Therefore, further studies are needed to understand the mechanism of action of LE, and its regulation of embryo size in rice grains.

With the increase in the embryo size of giant embryo mutants (*le* < *ge* < *ge*^s^), the GABA content of brown rice also increased by 150%, 400%, and 850% compared with wild type, respectively. In addition to GABA content, other nutrients and micronutrients such as protein, lipid, vitamin B1 and vitamin E were also higher in *ge*^*s*^ and *le* mutant compared to normal rice (Koh et al. 1993; Kim and Kim 2013; Chung et al. 2014a). Giant embryo mutants with high nutritional content are very useful breeding materials to improve the nutritional value of rice. Therefore, the creation and discovery of new genetic variation controlling embryo size is very important for breeding cultivars with high nutritional value. We used our three giant embryo mutants to breed high nutritional rice cultivar and had registered four cultivars to plant variety protection national list in Korea (soenong8 and seonong16 derived from *ge*; seonong14 derived from *ge*^*s*^; seonong17 derived from *le*).

Enlarged embryo size and easy embryo detachment are desirable traits of rice cultivars used for bran or bran oil production. However, High EER giant embryo mutants such as *ge* and *ge*^*s*^ are can’t be used to increase the nutritional value of milled or polished rice, because most of the embryo are separated from the rice grain during the milling process. Therefore, most of giant embryo rice cultivars are supplied as unpolished rice (brown rice) to customers. On the other hand, seonong17, a cultivar derived from *le* mutant with relatively low EER, showed 88% of embryo residual rate with developed milling process (Kim and Kim 2013). This result implicates that the *le* mutant, with mildly enlarged embryo, is expected to serve as an appropriate breeding material for improving the nutritional quality of milled rice because large part of embryo remain attached to the endosperm even after milling.

Generally, giant embryo mutants exhibit problematic germination and seedling growth. For example, the *go* mutant shows pleiotropic phenotypes, including viviparous seed, shortened plastochron, and conversion of panicle primary branches to vegetative shoots, in addition to the enlarged embryo phenotype (Kawakatsu et al. 2009). Similarly, the *ge* mutant shows abnormal seedling growth characterized by the production of malformed leaves and dwarfism, which eventually result in death (Yang et al. 2013). By contrast, the *le* mutant showed normal germination rate and seedling emergence (data not shown). Besides, Seonong17 is effective in lowering body fat and has high hypolipidemic and antioxidant activities (Chung et al. 2014b, 2016). These observations suggest that the *le* mutant is a highly valuable genetic resource for improving the nutritional quality of rice.

Overall, we identified a new gene, *LE,* responsible for the enlarged embryo phenotype of the *le* mutant of rice, and characterized the potential of the *le* mutant as a breeding material. The embryo of *le* mutant was only mildly enlarged comparing to the embryos of *ge* and *ge*^*s*^ mutants. *LE* encodes a C3HC4-type RING finger protein, expecting new molecular mechanism to control embryo size. Although further investigations are needed to understand the function of *LE*, this study provides novel insights into the genetic mechanism of embryo development in rice and introduces new breeding material for high nutritional rice cultivars with large embryos.

## Conclusion

The rice *le* mutant showed a mild enlargement of embryo and increased GABA content compared with wild type. Through allelism tests and sequence analysis, we showed that the enlarged embryo phenotype of *le* mutant was not controlled by previously reported genes regulating embryo size but by *LE*, a new gene controlling embryo size. The *LE* gene encodes a C3HC4-type RING finger protein. The disruption of the RING domain led to the enlargement of embryo size, possibly due to the increase in size of scutellar parenchyma cells. *LE* was highly expressed during the late stage of seed development. These results extend our understanding of embryo development in rice and facilitate the breeding of new giant embryo rice cultivars.

## Methods

### Plant Materials

Three giant embryo mutants were generated from a fertilized egg cell of *japonica* cv. HC treated with *N*-methyl-*N*-nitrosourea and designated as *le*, *ge*, and *ge*^*s*^, in ascending order of embryo size (Kim et al. 1991, 1992; *ge*^*m*^ which is nomenclature of the mutant least altered embryo size was renamed as *le* to avoid confusion with alleles of *GE* gene). Wild-type *japonica* cv. HC was used as a control in phenotype analysis. The F_2_ seeds derived from a cross between *le* mutant and HC were used to calculate the segregation ratio. Map-based cloning was conducted using F_2_ and F_3_ populations derived from a cross between *le* mutant and cv. Hangangchal1 (*Tongil*-type). All plant materials were grown under normal cultivation conditions in the experimental paddy field of the Seoul National University, Suwon, Korea.

### Phenotypic Analysis

Dimensions of rice grains were measured from images captured under a microscope with HD’MEASURE software (HANA Vision, Korea). To measure ENW, EMW, and EER, embryos and endosperms were dissected from husked seeds (14% water content) and weighed separately using an electronic balance (CAS, USA). Agronomic traits such as days to heading (DTH), culm length (CL), panicle length (PL), panicle number (PN), spikelet number per panicle (SPP), and spikelet fertility (SF) were also measured to compare morphological differences between HC and giant embryo mutants. All phenotypic data collected from HC and mutants were statistically analyzed using IBM SPSS Statistics software version 24 (IBM, USA).

### Histological Analysis

Seeds of HC and giant embryo mutants harvested at 20 DAP were fixed in formalin-acetic acid-alcohol (FAA) fixative (3.7% formaldehyde, 5% acetic acid, and 50% ethanol) for 24 h at 4 °C. Seeds were dehydrated by soaking them in a series of ethanol solutions of increased concentration for 2 h per each solution. After final dehydration using 100% ethanol, seeds were cleaned using a series of cleaning solution with progressively decreasing ethanol concentration and increasing concentration of histoclear, followed by soaking in 100% histoclear solution overnight. For paraffin infiltration, Paraplast® (Sigma, USA) was gradually added to histoclear solution at 60 °C. Finally, seeds were stored in 100% melted paraffin for 24 h at 60 °C. The paraffin-infiltrated samples were embedded in an embed block and cut into 10 μm sections with an *HM 340 E* Rotary Microtome (Microm Lab, Germany). The sections were stained with 1% safranin O solution (1% safranin O and 30% ethanol) and examined under CX31 Microscope (Olympus, Japan).

### Quantification of GABA Content

Fifty milligrams of finely ground brown rice powder was homogenized with 1 ml of 0.01 N HCl prepared in 25% acetonitrile and centrifuged at 13,000 rpm for 3 min. The supernatant was filtered through a 0.2 μm syringe filter. The clear supernatant was derivatized using EZ:FAAST Kit (Phenomenex, USA) and subjected to gas chromatography (GC) using a GC-2010 gas chromatograph (GC-2010, Japan) equipped with a flame ionization detector (FID) and a ZB-5 capillary column (30 m × 0.25 mm; 0.25 μm diameter) for the analysis of GABA. Each sample was tested four times.

### Allelism Test and Sequence Analysis

F_1_ and F_2_ seeds derived from reciprocal crosses among HC, and each of the three mutants, *le*, *ge*, and *ge*^s^, were used for genetic analysis. F_2_ seeds produced from each cross were classified according to the embryo size, and chi-square (χ^2^) test was performed to detect statistically significant differences. Two overlapping DNA fragments spanning the *GE* ORF (Nagasawa et al. 2013) were PCR amplified and sequenced with ABI Prism 3730 XL DNA Analyzer (PE Applied Biosystems, USA). The sequences of HC and three giant embryo mutants were aligned using CodonCode Aligner software (CodonCode Corporation, USA) and analyzed. Co-segregation test was conducted to detect SNP using CAPS and dCAPS markers (Additional file 1: Table S1).

### Map-Based Cloning of *LE*

F_2_ seeds derived from a cross between *le* mutant and Hangangchal 1 were classified into mutant type and wild type based on the embryo size. Twenty mutant type F_2_ seeds and 20 wild type F_2_ seeds were selected to prepare DNA bulks. Mutant and wild DNA bulks were subjected to BSA using 66 sequence-tagged-site (STS) markers (Michelmore et al. 1991; Chin et al. 2007). For fine mapping the *LE* gene, 397 F_2_ and 857 F_3_ plants were used, and seven new STS markers were designed using Primer3 version 0.4.0 (http://bioinfo.ut.ee/primer3-0.4.0) based on polymorphisms between sequences of *indica* and *japonica* rice (https://blast.ncbi.nlm.nih.gov and http://gramene.org). Primer sequences used in this study are listed in Additional file 1: Table S1.

### Candidate Gene Analysis

To identify nucleotide polymorphisms in candidate genes, DNA fragments of genes were PCR amplified from HC and *le* mutant using gene-specific primers (Additional file 1: Table S1) and purified using Gel & PCR Purification Kit (Inclone, Korea). The purified PCR products were cloned into pGEM-T Easy vector (Promega, USA) and transformed into *Escherichia coli* strain DH5α. Plasmid DNA was isolated from the transformed colonies using Plasmid MiniPrep Kit (Inclone, Korea) and sequenced using an ABI Prism 3730 XL DNA Analyzer (PE Applied Biosystems, USA). DNA sequencing data were aligned using CodonCode Aligner software (CodonCode Corporation, USA).

### Multiple Sequence Alignment and Phylogenetic Analysis

The amino acid sequences with high similarity to LE were downloaded from the Universal Protein Resource (UniProt, http://www.uniprot.org). Multiple sequence alignment was carried out using Probcons, and background color shading was applied with Jalview using the percent identity scheme. Details of protein sequences used in this study are provided in Additional file 2: Figure S1. Transmembrane motifs were predicted using TMHMM server v. 2.0 (http://www.cbs.dtu.dk/services/TMHMM/).

### RNA Extraction and qRT-PCR Analysis

Total RNA was extracted from leaf, stem, and root tissues collected at heading time, and from seeds 5 and 20 DAP, using Hybrid-R™ RNA Purification Kit (GeneAll, Korea). All RNA samples were treated with RNase-free Recombinant DNase Ι (Takara Bio, Japan) to eliminate genomic DNA contamination. First-strand cDNA was synthesized using M-MLV reverse transcriptase (Promega, USA). qRT-PCR was performed using SYBR *Premix Ex Taq* (Takara, Japan) on a CFX96™ Real-Time PCR Detection System (Bio-Rad, USA), according to the manufacturer’s instructions. Primers used for qRT-PCR analysis are listed in Additional file 1: Table S1. Expression levels of *LE* were normalized relative to *UBIQUITIN5*, a housekeeping gene.

### GUS Staining

GUS staining was carried out as previously described (Jefferson et al. 1987). The GUS staining solution was vacuum-infiltrated into various organ samples for 10 min. All samples were submerged in the staining solution and incubated overnight at 37 °C. This was followed by incubation in 90% ethanol overnight at room temperature to bleach the chlorophyll.

### Vector Construction and Transformation

For the generation of RNAi and *proLE*::GUS transgenic plants, the 3′ untranslated region (3′ UTR; + 6925 to + 7215 bp) and promoter region (− 2135 to − 1 bp) of *LE* were PCR amplified and cloned into pH7GWIWG2(II) and pHGWFS7, respectively, using Gateway® BP and LR Clonase™ II enzyme mixes (Invitrogen, USA). The final constructs were introduced into the *japonica* cv. Dongjin via *Agrobacterium*-mediated transformation using the strain LBA4404 as described (Nishimura et al. 2007) with slight modifications. Primers used for cloning PCR products are listed in Additional file 1: Table S1.

## Additional files

Additional file 1: **Table S1.** Agronomic traits of HC three giant embryo mutants. DTH, days to heading; CL, culm length; PL, panicle length; PN, panicle number; SPP, spikelet per panicle; SF, spikelet fertility; GL, grain length; GW, grain width; LWR, length-width ratio. The means followed by lowercase letters are significantly different (*p* < 0.05, ANOVA) according to Duncan test. (XLSX 10 KB)

Additional file 2: **Figure S1.** Types of segregated F_2_ seed derived from cross-combinations of *le*/*ge* (a) and *le*/*ge*^*s*^ (b). (PDF 96 KB)

Additional file 3: **Figure S2.** Multiple sequence alignment of LE orthologs. Asterisk represents the location of amino acid substitution caused by one base deletion in le mutant. Substituted amino acid residues by frameshift are highlighted by red background shading. Predicted transmembrane motifs and the C3HC4-type RING finger domain are underlined with blue and red lines, respectively. Conserved residues in the C3HC4-type RING finger domain are enclosed within black open boxes. *Os*, *Oryza sativa*; *Ta*, *Triticum aestivum*; *Hv*, *Hordeum vulgare*; *Zm*, *Zea mays*; *At*, *Arabidopsis thaliana*; *Br*, *Brassica rapa*; *Gm*, *Glycine max*. (PDF 531 KB)

Additional file 4: **Figure S3.** Co-segregation test on F_2_ individuals and various rice genotypes. a Co-segregation test on F_2_ individuals derived from the cross between HC/*le* mutant. b Co-segregation test on other varieties and *O.nivara*. 1, Hwacheong; 2, Hwaseonchal, 3. Ilpum, 4. Unkwang, 5. Hapcheon, 6. Nipponbare, 7. Dasan, 8. Hangangchal 1ho, 9. IR36, 10. IR64, 11. IR56, 12. IR21015, 13. *O.nivara*. (PDF 23 KB)

Additional file 5: Information of the primers used in this study. (XLSX 11 KB)

Additional file 6: Information of LE orthologs used in multiple sequence alignment. (XLSX 9 KB)

## Abbreviations

BSA: Bulked segregant analysis
CL: Culm length
DAP: Days after pollination
EER: Embryo to endosperm ratio
EL: Embryo length
EMW: Embryo weight
ENW: Endosperm weight
GE: Giant embryo
GL: Grain length
GUS: β-glucuronidase
GW: Grain width
HC: Hwacheong
LE: Large embryo
LWR: Length to width ratio
NILs: Near-isogenic lines
ORF: Open reading frame
qRT PCR: Quantitative real time PCR
RNAi: dsRNA-mediated interference
TGW: Thousand-grain weight
