# Supplementary figures and images for: Identification and Characterization of LARGE EMBRYO, a New Gene Controlling Embryo Size in Rice (Oryza sativa L.)
Source: Rice (N Y). 2019 Apr 11;12:22. doi: 10.1186/s12284-019-0277-y (PMC6458227; doi:10.1186/s12284-019-0277-y)

## Additional file 2: Figure S1

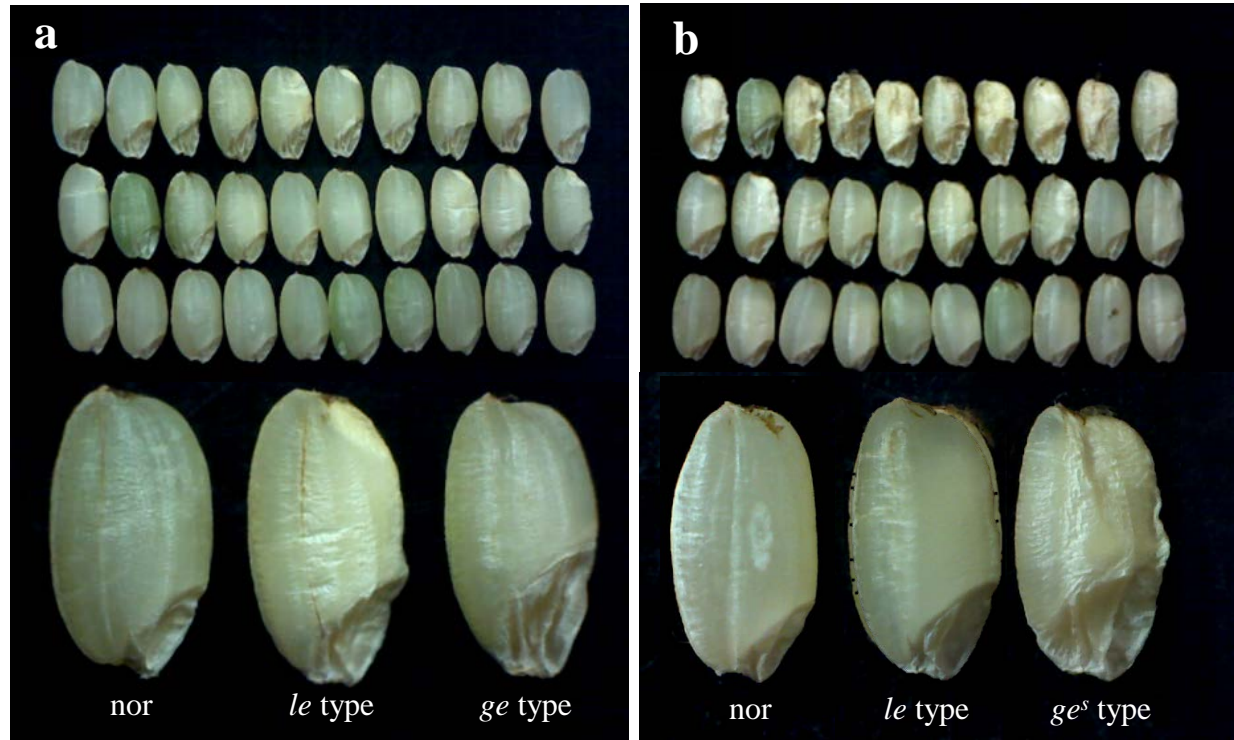

Supplement: Supplementary file 2 — Figure S1. Types of segregated F2 seed derived from cross-combinations of le/ge (a) and le/ges (b). (PDF 96 KB) [file 12284_2019_277_MOESM2_ESM.pdf]

Additional file 4: Figure S3

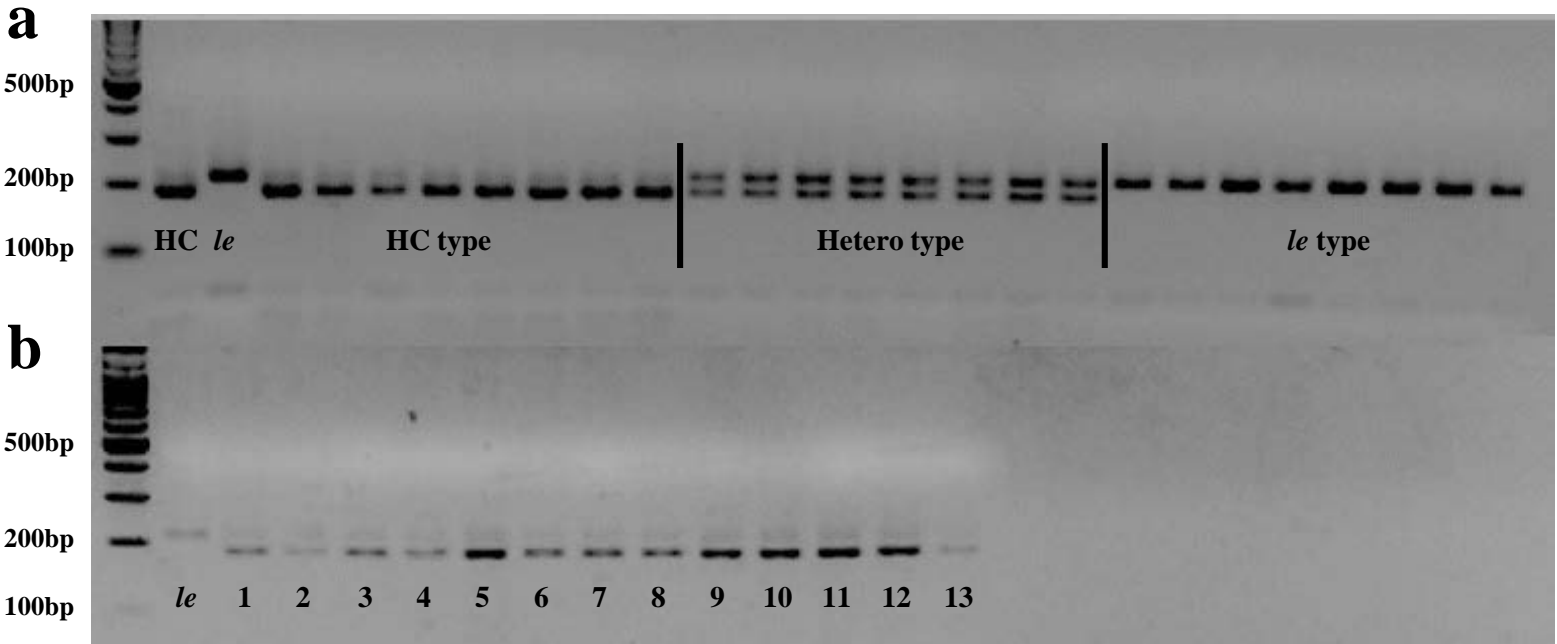

Supplement: Supplementary file 4 — Figure S3. Co-segregation test on F2 individuals and various rice genotypes. a Co-segregation test on F2 individuals derived from the cross between HC/le mutant. b Co-segregation test on other varieties and O.nivara. 1, Hwacheong; 2, Hwaseonchal, 3. Ilpum, 4. Unkwang, 5. Hapcheon, 6. Nipponbare, 7. Dasan, 8. Hangangchal 1ho, 9. IR36, 10. IR64, 11. IR56, 12. IR21015, 13. O.nivara. (PDF 23 KB) [file 12284_2019_277_MOESM4_ESM.pdf]
