# Supplementary material for: Identification and Characterization of LARGE EMBRYO, a New Gene Controlling Embryo Size in Rice (Oryza sativa L.)
Source: Rice (N Y). 2019 Apr 11;12:22. doi: 10.1186/s12284-019-0277-y (PMC6458227; doi:10.1186/s12284-019-0277-y)

Additional file 3: Figure S2

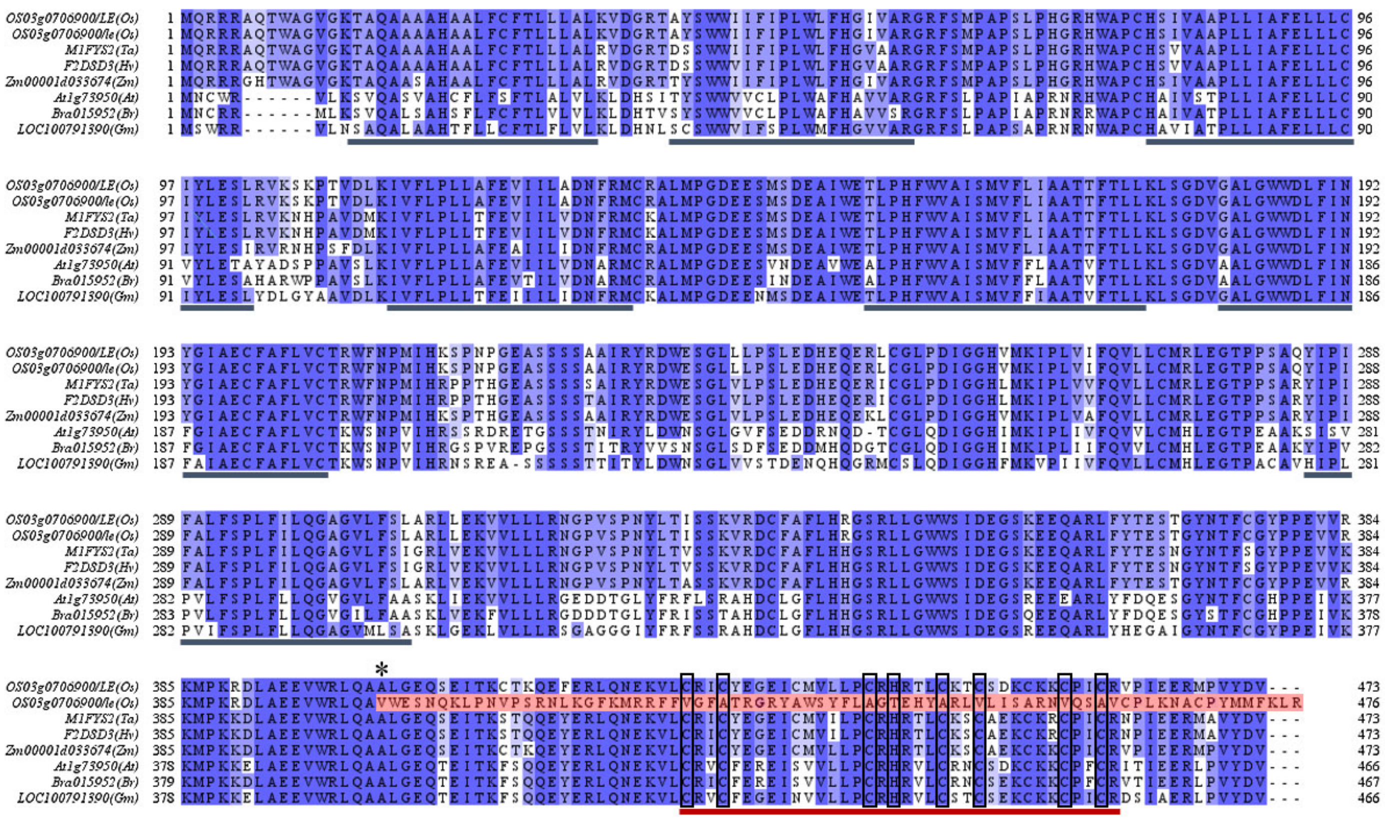

Supplement: Supplementary file 3 — Figure S2. Multiple sequence alignment of LE orthologs. Asterisk represents the location of amino acid substitution caused by one base deletion in le mutant. Substituted amino acid residues by frameshift are highlighted by red background shading. Predicted transmembrane motifs and the C3HC4-type RING finger domain are underlined with blue and red lines, respectively. Conserved residues in the C3HC4-type RING finger domain are enclosed within black open boxes. Os, Oryza sativa; Ta, Triticum aestivum; Hv, Hordeum vulgare; Zm, Zea mays; At, Arabidopsis thaliana; Br, Brassica rapa; Gm, Glycine max. (PDF 531 KB) [file 12284_2019_277_MOESM3_ESM.pdf]
